# Editorial: Omics applications for pathogen control and disease resistance

**DOI:** 10.3389/fpls.2026.1859438

**Published:** 2026-05-19

**Authors:** Molemi Rauwane, Rudo Ngara, Chioma Blaise Chikere, Khayalethu Ntushelo

**Affiliations:** 1 Department of Biotechnology and Food Technology, University of Johannesburg, Doornfontein, South Africa; 2 Department of Plant Sciences, University of the Free State, Phuthaditjhaba, South Africa; 3 Department of Microbiology, Faculty of Science, University of Port Harcourt, Port Harcourt, Rivers State, Nigeria; 4 Department of Agriculture and Animal Health, University of South Africa, Florida, South Africa

**Keywords:** control, disease, omics, plant, resistance

Recent advances in omics technologies have substantially boosted research in genome, transcriptome, proteome and metabolome characterization. This Research Topic aimed to collate articles on the applications of omics in studying pathogen control and disease resistance. Primarily the topic attracted interest from authors who studied transcriptomic and metabolomic responses of the plant to pathogen infection. There was also pathogen genome characterization, focusing on a tomato brown rugose virus (ToBRV) isolate from Mexico. Genomic analysis of the ToBRV isolate from Mexico (PQ628197) was conducted to understand the virus’s adaptability and transmission dynamics (Zamora-Macorra et al.,). The results revealed high genetic similarity between the isolate and 100 other global ToBRV genomes, highlighting the stability of ToBRV across geographical regions. Host-susceptibility bioassays revealed that the Mexican ToBRV isolate replicated exclusively in solanaceous hosts and not in their cucurbit counterparts, with tobacco, tomatillo, and eggplant identified as new susceptible hosts. Virus-infected tobacco seeds exhibited reduced germination rates and viability compared with uninfected controls, further illustrating the potential effects of viral infection on the reproductive success of its hosts. Overall, this study expands our understanding of the molecular basis of ToBRV’s adaptability, potential transmission pathways, host range across cultivated and wild species, and its potential biosecurity risk due to seed-borne transmission (Zamora-Macorra et al.,). Scrutinizing studies on pathogen identification and disease control strategies using genomic tools Ogbuji and Agogbua, wrote a review article which reported on the importance of plant pathogen identification and control strategies using genomics tools. Genomic tools such as next generation sequencing, can accelerate diagnostic workflows and enhance precision by relying on the detection of pathogen-specific genetic markers. In addition, genome editing technologies have been shown to provide pathways to improve plant resistance against infections by targeting susceptibility genes, which pathogens use to initiate infection and replicate within the host plant. According to the authors, genomic tools such as draft genome sequence of pathogens and their host plants, and the gene editing tool, CRISPR-cas9 offer researchers the opportunity to discover and incorporate resistance in host plants to increase plant yield. This study highlighted efficiency of genomics and genome editing tools, along with the quality and volume of data they generate to answer important questions in science.

Yin et al. observed increase in phenylalanine deaminase, catalase, and peroxidase enzymes in *Polygonatum cyrtonema in* response to *Botrytis deweyae* infection. Likewise key disease response pathways were reactive with a concomitant upregulation of various genes some involved in transcription regulation. Additionally, the upregulation of α-linolenic acid and jasmonic acid synthesis were observed in response to *B. deweyae*, suggesting the potential activation of jasmonic acid-dependent induced systemic resistance against *B. deweyae.* These findings provided insights that may support the development of disease-resistant cultivars or biostimulant strategies for *P. cyrtonema* and related medicinal plants.

Le Clerc et al. reported the upregulation of six previously identified genes, *PR1, PR4, PR8, POX, PECT*, and *WRKY* post treatment with the biopesticides Sonata^®^ and Bion^®^ in carrots. The two biopesticides were able to activate the systemic acquired resistance pathway as evidenced by the overexpression of PR protein genes. The application of biopesticide products enabled the protection of carrots against Alternaria leaf blight. Because the study assessed responses of different varieties it was observed that the plant molecular responses were also variety dependent.

Makhumbila et al. investigated the use of RNA-Seq in assessing gene expression patterns of common bean response to the rust pathogen *Uromyces appendiculatus* at different time points. The study reported the upregulation/downregulation of stress responsive genes such as those which code for heat shock proteins, receptor-like kinases, cytochrome monooxygenases and terpene synthases, among others, suggesting different response patterns. In addition, genes *RPS2, CAR1* and *DM2H* were among the identified potential biomarkers associated with *U. appendiculatus* resistance. This study highlighted that RNA-Seq approaches can be used for biomarker discovery and possible development of resistant varieties in response to pathogen infection.

Abdel-Wahab et al. demonstrated that treatment with nanoparticles reduced nematode proliferation in pomegranate-planted soil with a matching heightening of various morpho-biochemical parameters which included leaf area, levels of photosynthetic pigments (chlorophyll *a*, *b*, and carotenoids), total antioxidants, thiol compounds [glutathione (GSH), non-protein thiols (NPTs), protein thiols (PTs)], and flavonoids in the plant. There was a downward effect on accumulation of malondialdehyde (MDA) and proline, and stress markers, in pomegranate plants infected with nematodes. Nanoparticle treatments did not affect the production of phenolic compounds. It was therefore concluded that nanoparticle effect partially depends on the increased production of photosynthetic pigments, thiol compounds, and flavonoids.

In their research, Mahlangu et al. revealed a differential response of two potato cultivars, Sifra and Valor, after inoculation with a bacterial pathogen, *Pseudomonas syringae*. Three intervals of leaf metabolome assessment, namely, 24, 48 and 72 hours, after inoculation showed a varied response defined by timing of response and the suites of metabolites upregulated during each of the three analysis windows. It was concluded that genotype was instrumental in either the timing of response to inoculation or in the metabolites deployed as a response to the pathogen.

In identifying key genes governing *Verticillium* wilt resistance in cotton using machine learning and weighted gene co-expression network analysis, Lei at al. discovered a robust set of core genes involved in pathogen recognition (*GhRLP6*), calcium signaling (*GhCIPK6*, *GhCBP60A*), hormone response, and secondary metabolism (*GhF3’H*) and this provided novel insights into the spatiotemporal regulation of immune responses in cotton.

Lin et al. work uncovered that pepper mild mottle virus induced more pronounced mosaic symptoms and higher viral accumulation levels in the susceptible pepper line 21C241 compared to the resistant line 21C385. Four hundred and sixty two and 401 differentially expressed genes were discovered in the systemically infected leaves of the susceptible and resistant lines, respectively, when compared to their healthy counterparts. Most of the discovered differentially expressed genes control photosynthesis and the biosynthesis of secondary metabolites, with 28 differentially expressed genes exhibiting distinct expression patterns between the two lines.

Notably, the expression level of the chlorophyll *a-b* binding protein 13 (CAB13) was significantly upregulated in the resistant line 21C385 following viral infection. Functional analysis through silencing of *CAB13* in pepper and *Nicotiana benthamiana* demonstrated a reduction in virus accumulation, suggesting that CAB13 plays a positive role in facilitating pepper mild mottle virus infection in pepper plants. This study demonstrated genotype-specific responses and overall, it revealed molecular mechanisms in gene expression governed by various light-chlorophyl dynamics.

Lerenard et al. (showed that plant defense compounds can enhance antagonistic effects against *Alternaria brassicicola* of seed-associated fungi isolated from wild Brassicaceae and recommend attempts to further understand relationships between host plants, pathogens and fungal endophytes.

The 10 studies added the much-needed value in plant-pathogen relations studies and will serve as a platform for future studies. They also solidified previous findings that pathogens induce expression of defense genes and activate defense pathways. Metabolite responses complete the disease response machinery. These studies provided insights on how disease resistance can be incorporated in the several crops studied.

